# Children's and adolescents' lifestyle factors associated with physical activity in five Mediterranean countries: the DELICIOUS project

**DOI:** 10.3389/fpubh.2025.1654645

**Published:** 2025-09-24

**Authors:** Alice Rosi, Francesca Scazzina, Maria Antonieta Touriz Bonifaz, Francesca Giampieri, Achraf Ammar, Khaled Trabelsi, Osama Abdelkarim, Mohamed Aly, Evelyn Frias-Toral, Juancho Pons, Laura Vázquez-Araújo, Josep Alemany-Iturriaga, Lorenzo Monasta, Nunzia Decembrino, Ana Mata, Adrián Chacón, Pablo Busó, Giuseppe Grosso

**Affiliations:** ^1^Human Nutrition Unit, Department of Food and Drug, University of Parma, Parma, Italy; ^2^Facultad de Ciencias de la Salud, Universidad Católica de Santiago de Guayaquil, Guayaquil, Ecuador; ^3^Facultad de Ciencias Médicas, Universidad de Guayaquil, Guayaquil, Ecuador; ^4^Department of Clinical Sciences, Università Politecnica delle Marche, Ancona, Italy; ^5^Research Group on Food, Nutritional Biochemistry and Health, Universidad Europea del Atlántico, Santander, Spain; ^6^Joint Laboratory on Food Science, Nutrition, and Intelligent Processing of Foods, Polytechnic University of Marche Italy, Universidad Europea del Atlántico Spain and Jiangsu University China, at Polytechnic University of Marche, Ancona, Italy; ^7^International Research Center for Food Nutrition and Safety, Jiangsu University, Zhenjiang, China; ^8^Department of Training and Movement Science, Institute of Sport Science, Johannes Gutenberg-University Mainz, Mainz, Germany; ^9^Research Laboratory, Molecular Bases of Human Pathology, Faculty of Medicine, University of Sfax, Sfax, Tunisia; ^10^Research Laboratory: Education, Motricity, Sport and Health, EM2S, LR19JS01, High Institute of Sport and Physical Education of Sfax, University of Sfax, Sfax, Tunisia; ^11^Department of Movement Sciences and Sports Training, School of Sport Science, The University of Jordan, Amman, Jordan; ^12^Faculty of Sport Sciences, Assiut University, Assiut, Egypt; ^13^ESLSCA University Egypt, Giza, Egypt; ^14^Escuela de Medicina, Universidad Espíritu Santo, Samborondón, Ecuador; ^15^Division of Research, Texas State University, San Marcos, TX, United States; ^16^Editorial Luis Vives (EDELVIVES), Carretera de Madrid, Zaragoza, Spain; ^17^BCC Innovation, Technology Center in Gastronomy, Basque Culinary Center, Donostia-San Sebastián, Spain; ^18^Basque Culinary Center, Faculty of Gastronomic Sciences, Mondragon Unibertsitatea, Donostia-San Sebastián, Spain; ^19^Department of Health, Nutrition and Sport, Universidad Internacional Iberoamericana, Campeche, Mexico; ^20^Universidade Internacional do Cuanza, Cuito, Bié, Angola; ^21^Institute for Maternal and Child Health – IRCCS Burlo Garofolo, Trieste, Italy; ^22^Neonatal Intensive Care Unit, University Hospital “Policlinico-San Marco” Catania, Integrated Department for Maternal and Child's Health Protection, Catania, Italy; ^23^Technological Institute for Children's Products & Leisure AIJU, Alicante, Spain; ^24^Department of Biomedical and Biotechnological Sciences, University of Catania, Catania, Italy; ^25^Center for Human Nutrition and Mediterranean Foods (NUTREA), University of Catania, Catania, Italy

**Keywords:** physical activity, lifestyle, children, adolescents, Mediterranean area

## Abstract

**Background:**

Physical activity in children and adolescents represents one of the most important lifestyle factors to determine current and future health.

**Aim:**

The aim of the study is to assess the lifestyle and dietary factors linked to physical activity in younger populations across five countries in the Mediterranean region.

**Design:**

A total of 2,011 parents of children and adolescents (age range 6–17 years) participating to a preliminary survey of the DELICIOUS project were investigated to determine children's adequate physical activity level (identified using the short form of the international physical activity questionnaire) as well as diet quality parameters [measured as Youth-Healthy Eating Index (Y-HEI)] and eating and lifestyle factors (i.e., meal habits, sleep duration, screen time, etc.). Logistic regression analyses were performed to assess the odds ratios (ORs) and 95% confidence intervals (CIs) for the associations between variables of interest.

**Results:**

Younger children of younger parents currently working had higher rates and probability to have adequate physical activity. Multivariate analysis showed that children and adolescents who had breakfast (OR = 1.88, 95% CI: 1.38, 2.56) and often ate with their family (OR = 1.80, 95% CI: 0.90, 3.61) were more likely to have an adequate level of physical activity. Children and adolescents who reported a sleep duration (8–10 h) closest to the recommended one were significantly more likely to achieve adequate levels of physical activity (OR = 1.88, 95% CI: 1.38, 2.56). Conversely, those with more than 4 h of daily screen time were less likely to engage in adequate physical activity (OR = 0.77, 95% CI: 0.54, 1.10). Furthermore, children and adolescents in the highest tertile of YEHI scores showed a 60% greater likelihood of engaging in adequate physical activity (OR = 1.60, 95% CI: 1.27, 2.01).

**Conclusion:**

These results emphasize the importance of promoting healthy diet and lifestyle habits, including structured and high quality shared meals, sufficient sleep, and screen time moderation, as key strategies to support active behaviors in younger populations. Future interventions should focus on reinforcing these behaviors through parental guidance and community-based initiatives to foster lifelong healthy habits.

## 1 Introduction

Physical activity offers numerous benefits to the human body, including prevention of diseases and treatment and rehabilitation in certain conditions (1). The existing evidence suggests a well-defined dose-response relationship between physical activity and overall health (2). Regular engagement in physical activity has been associated with a significant reduction in the risk of premature mortality and is an established means of reducing the risks for several chronic non-communicable diseases, including cardiovascular disease, metabolic disorders, and certain cancers (3). It also greatly contributes to improving cardiorespiratory endurance and muscular strength, making the heart and muscles more efficient in their daily functioning (4). Physical activity supports the development and maintenance of stronger bones, reducing the risk of bone fragility or conditions like osteoporosis as age progresses (5). Also, physical activity may play a role in the prevention not only of physical illnesses but also of certain psychiatric disorders such as depressive and anxiety disorders (6). In this context, it has been shown that reducing inactivity by just 10% could prevent over 530,000 deaths per year, and a 25% reduction could save over 1.3 million lives (7).

When practiced during childhood, physical activity presents other significant benefits, as it tends to continue throughout life (8). This is especially important, as physically active adults tend to lead healthier lives, both physically and emotionally (9). It provides them with the chance to engage in playful activities, enjoy themselves, and discover their surroundings. Physical activity supports the development of motor skills and boosts energy expenditure (10). Moreover, physically active individuals engage in overall healthier lifestyles while the sedentary adolescent population exhibit a poorer lifestyle, including increased screen time, time spent studying, as well as depression and smoking (11). The World Health Organization recommends that children and adolescents aged 6–17 years engage in at least 60 min of moderate-to-vigorous physical activity (MVPA), with the majority being aerobic exercises to promote overall health and well being (12). Additionally, at least 3 days per week, activities should include vigorous-intensity exercises and muscle- and bone-strengthening activities such as jumping, climbing, or resistance training (13). Given the overwhelming evidence supporting the physical, cognitive, and mental health benefits of regular physical activity, promoting an active lifestyle remains a key public health priority (14).

Despite the numerous and well-documented benefits of physical activity on health, many people continue to lead a sedentary lifestyle. About 30% of adults and 80% of adolescents do not currently meet the recommended levels of physical activity, with lower rates registered in some Mediterranean countries (15). Sedentary living is associated with several factors, including the increasing use of technology, long hours spent in the office or at school, and a lack of time or motivation to engage in regular physical activity. Recent findings from the Global Burden of Disease study reveal a concerning increase in the impact of low physical activity on mortality and disability. Since 1990, its contribution to deaths and disability-adjusted life years (DALYs) has risen by over 80%, reaching ~1 million deaths and 16 million DALYs in 2019 (16). Such trends are of particular interest in the Mediterranean region, in which poor physical activity have been reported to have particularly high prevalence, especially among younger individuals (16). The Mediterranean culture has historically been characterized by an active lifestyle thanks to working time in the fields and time spent out of home in convivial activities; however, such a lifestyle is long abandoned giving place to more modern habits, with particular matter for younger generations spending longer time on screens, scarcely participating in school activities, and potentially embracing in isolating behaviors associated with poor physical activity. These alarming figures underscore the urgent need for a comprehensive understanding of the factors driving this trend, particularly among younger generations. Identifying key determinants of physical activity engagement is essential for developing targeted public health interventions that promote active lifestyles, improve overall health outcomes, and ultimately reduce the long-term burden on healthcare systems. The aim of the study is to assess the lifestyle and dietary factors linked to physical activity in younger populations across five countries in the Mediterranean region.

## 2 Methods

### 2.1 Study design and population

The present study is based on a cross-sectional evaluation conducted within the scope of the DELICIOUS project, which is funded by the European Union (UnDErstanding consumer food choices and promotion of healthy and sustainable Mediterranean Diet and LIfestyle in Children and adolescents through behavIOUral change actionS) (17). This research involved a consumer survey targeting parents of children and adolescents aged 6–17 years across five Mediterranean countries: Italy, Spain, Portugal, Egypt, and Lebanon. Participants were enlisted based on their voluntary agreement to be part of a consumer database. Informed by recent studies with similar goals in Mediterranean nations (18–20), a goal of 400 participants was set for each country in the region. The data were collected via an online survey, leading to the recruitment of a total of 2,011 participants. The study protocol was approved by the ethics committee of Mondragon University (no. IEB-20230704). All processes were carried out in accordance with the Declaration of Helsinki (1989) established by the World Medical Association, and every participant signed an informed consent form before taking part in the research.

### 2.2 Data collection

Information regarding participants' demographic and lifestyle characteristics was gathered in 2019. Data for parents included sex, age, educational attainment, and employment status, while for children and adolescents, details on gender, age, and anthropometric measurements were collected. The ages of children and adolescents were grouped into four categories: 6–8, 9–11, 12–14, and 15–17 years. Parental education was classified into three levels: low (primary education), medium (secondary education), and high (tertiary education). Employment status was noted as either unemployed or employed. Children's and adolescents' BMI was calculated using their height and weight, and categorized based on percentile ranges from the CDC growth charts for individuals aged 2–19 years (21). BMI classifications included normal weight (5th−84th percentile), overweight (85th−94th percentile), and obese (≥95th percentile).

Participants responded to a series of questions about their children's eating behaviors, covering aspects such as breakfast routines, dining location, meal frequency, eating companions, and preferences for home-cooked vs. advertised foods. Additionally, screen time was categorized into three groups: <2 h per day, 2–4 h per day, and more than 4 h per day. Participants were also asked to report the average number of hours their children slept each night. Based on the National Sleep Foundation's guidelines, sleep duration was classified as sufficient (8–10 h) or insufficient (either shorter or longer than this range) (22).

### 2.3 Dietary intake

Parents were asked to provide information about their children's food consumption over the past 24 h, detailing daily dietary intake. Responses included predefined categories based on meal types, along with an option to add other foods not listed. To evaluate weekly dietary patterns, questions focused on the frequency of key food group consumption. The dietary data were analyzed using the Youth Healthy Eating Index (Y-HEI), a tool designed to measure diet quality in older children and adolescents (23). This index assesses factors like fat, fiber, sodium, and added sugar intake, emphasizing food choices rather than precise nutrient calculations, making it suitable for younger populations. The Y-HEI comprises 13 components, including intake of whole grains, fruits, vegetables, dairy, meat, snacks, sugary beverages, fried foods, butter and margarine, visible animal fats, and lifestyle habits such as eating breakfast and sharing meals with family. Scores range from 0 to 100, with higher scores reflecting better diet quality. In this study, data on multivitamin use and visible fat intake were excluded, reducing the maximum score to 90.

### 2.4 Physical activity

Physical activity was evaluated by asking participants about the duration and intensity of their daily physical activity, if performed. Activity levels were assessed using the International Physical Activity Questionnaire-Short Form (IPAQ-SF), which gathers data on physical activity over the past 7 days (24). This includes information on the frequency and daily duration of walking, moderate-intensity, and vigorous-intensity activities. Considering WHO physical activity recommendations, children and adolescents (6–17 years) should engage in at least 60 min of moderate-to-vigorous physical activity (MVPA) daily, totaling 420 min per week. Accordingly, IPAQ-SF data were used to classify physical activity levels as adequate or inadequate based on these recommendations. Adequate physical activity was defined as accumulating at least 420 min of MVPA per week or meeting a threshold of ≥600 MET-min/week, though some studies propose ≥2,520 MET-min/week to better align with the WHO-recommended 60 min/day of MVPA. Inadequate physical activity was classified as engaging in <420 min of MVPA per week or accumulating <600 MET-min/week, indicating insufficient physical activity relative to WHO guidelines.

### 2.5 Statistical analysis

Categorical data are presented as counts and percentages, with group comparisons performed using the Chi-square test. Continuous data are reported as mean values with standard deviations (SDs), and differences between groups are analyzed using the Student's *t*-test. Logistic regression models were used to calculate odds ratios (ORs) and 95% confidence intervals (CIs) to explore the associations between various exposure variables (i.e., demographics, lifestyles, and dietary behaviors) as well as dietary factors and tertiles and a 1-SD increment in Y-HEI scores with adequate physical activity. The analyses of variables associated with adequate physical activity were multivariate adjusted by groups of demographics (age groups, sex, weight status, parent's age, parent's occupational level, parent's educational level, and area of living), lifestyles (breakfast habit, eating out of home, eating with family, eating alone, eating at school, eating advertised foods, eating home-made foods, screen time, and sleep duration), and eating behaviors (vegetables, fruit, cereals, dairy, meat, legumes, fish, nuts, whole grains, and sweets). Values of *p* < 0.05 were deemed as statistically significant. All analyses were performed using the statistical package IBM SPSS Statistics, version 28.0 (IBM Corp., Armonk, NY, USA).

## 3 Results

The demographic features of the parents, children, and adolescents involved in the study are shown in Table 1. After adjustment for all demographic variables, younger children of younger parents currently working had higher rates and probability to have adequate physical activity levels (Table 1).

**Table 1:** Demographic characteristics of parents and children and adolescents participating in the study according to adequate level of physical activity (*n* = 2,011).

	**Physical activity**			**Physical activity**	
	**Inadequate**	**Adequate**	***p*** **-Value**	**OR (95% CI)**	**OR (95% CI)** ^*^
**Age groups, (** ***n*** **, %)**			0.182		
Children (6–11 years)	462 (50.4)	585 (53.4)		1	1
Adolescents (12–17 years)	454 (49.6)	510 (46.6)		0.89 (0.74, 1.06)	0.79 (0.64, 0.98)
**Sex, (** ***n*** **, %)**			0.578		
Male	447 (48.8)	548 (50.0)		1	1
Female	469 (51.2)	547 (50.0)		0.95 (0.8, 1.13)	0.82 (0.67, 1.01)
**Weight status, (** ***n*** **, %)**			0.169		
Normal weight	503 (70.8)	584 (67.0)		1	1
Overweight	115 (16.2)	148 (17.0)		1.11 (0.85, 1.45)	0.95 (0.71, 1.27)
Obese	92 (13.0)	140 (16.1)		1.31 (0.98, 1.75)	1.12 (0.82, 1.52)
**Parent's age, (** ***n*** **, %)**			0.002		
<44 years	165 (18.0)	258 (23.6)		1	1
≥45 years	751 (82.0)	837 (76.4)		0.71 (0.57, 0.89)	0.70 (0.52, 0.94)
**Parent's occupational level, (** ***n*** **, %)**			<0.001		
Unemployed	665 (73.5)	868 (80.7)		1	1
Current working	240 (26.5)	208 (19.3)		0.66 (0.54, 0.82)	0.76 (0.58, 1.00)
**Parent's educational level, (** ***n*** **, %)**			<0.001		
Low	53 (6.1)	38 (3.6)		1	1
Medium	402 (45.9)	348 (32.9)		1.21 (0.78, 1.88)	1.03 (0.59, 1.81)
High	421 (48.1)	672 (63.5)		2.23 (1.44, 3.44)	1.61 (0.91, 2.83)
**Area of living, (** ***n*** **, %)**			0.068		
Urban	726 (79.3)	903 (82.5)		1	1
Rural	190 (20.7)	192 (17.5)		0.81 (0.65, 1.02)	0.98 (0.74, 1.28)

^*^Analyses were adjusted for all variables presented in the table.

Concerning lifestyle and eating behaviors (Table 2), multivariate analysis showed that children and adolescents who had breakfast were more likely to have an adequate level of physical activity (OR = 1.88, 95% CI: 1.38, 2.56; Table 2). Regarding eating location, the strongest association was observed among individuals who frequently ate with their family (OR = 1.80, 95% CI: 0.90, 3.61). Children and adolescents who reported a sleep duration (8–10 h) closest to the recommended one were significantly more likely to achieve adequate levels of physical activity (OR = 1.88, 95% CI: 1.38, 2.56). Conversely, adequate physical activity was associated with a moderate daily screen time (2–4 h; OR = 1.45, 95% CI: 1.19, 1.77).

**Table 2:** Lifestyle and eating behaviors of children and adolescents according to the adequate level of physical activity (*n* = 2,011).

	**Physical activity**			**Physical activity**	
	**Inadequate**	**Adequate**	***p*** **-Value**	**OR (95% CI)**	**OR (95% CI)** ^*^
**Breakfast habit, (** ***n*** **, %)**			<0.001		
Never/seldom	156 (17.0)	121 (11.1)		1	1
Often	127 (13.9)	221 (20.2)		2.24 (1.63, 3.10)	2.10 (1.50, 2.94)
Always	633 (69.1)	753 (68.8)		1.53 (1.18, 1.99)	1.88 (1.38, 2.56)
**Eating out of home, (** ***n*** **, %)**			<0.001		
Never	478 (52.2)	454 (41.5)		1	1
1 time	371 (40.5)	520 (47.5)		1.48 (1.23, 1.78)	1.30 (1.05, 1.61)
2 or more times	67 (7.3)	121 (11.1)		1.90 (1.37, 2.63)	1.67 (1.17, 2.39)
**Eating with family, (** ***n*** **, %)**			<0.001		
Seldom	25 (2.7)	16 (1.5)		1	1
Often	236 (25.8)	365 (33.3)		2.42 (1.26, 4.62)	1.80 (0.90, 3.61)
Daily	655 (71.5)	714 (65.2)		1.70 (0.90, 3.22)	1.35 (0.67, 2.68)
**Eating alone, (** ***n*** **, %)**			0.003		
Never/seldom	605 (25)	642 (58.6)		1	1
Often	246 (26.9)	351 (32.1)		1.34 (1.10, 1.64)	1.12 (0.86, 1.46)
Daily	65 (7.1)	102 (9.3)		1.48 (1.06, 2.06)	1.40 (0.98, 2.00)
**Eating at school, (** ***n*** **, %)**			<0.001		
Never/seldom	438 (47.8)	393 (35.9)		1	1
Often	246 (26.9)	376 (34.3)		1.70 (1.38, 2.10)	1.46 (1.16, 1.83)
Almost daily	232 (25.3)	326 (29.8)		1.57 (1.26, 1.94)	1.48 (1.18, 1.85)
**Eating advertised foods, (** ***n*** **, %)**			0.014		
No	497 (54.3)	534 (48.8)		1	1
Yes	419 (45.7)	561 (51.2)		1.25 (1.05, 1.49)	0.99 (0.80, 1.22)
**Eating home-made foods, (** ***n*** **, %)**			0.002		
Seldom	139 (15.2)	113 (10.3)		1	1
Often	378 (41.3)	513 (46.8)		1.67 (1.26, 2.21)	1.21 (0.89, 1.64)
Almost daily	399 (43.6)	469 (42.8)		1.45 (1.09, 1.92)	1.33 (0.99, 1.79)
**Screen time, (** ***n*** **, %)**			<0.001		
<2 h/day	557 (60.8)	574 (52.4)		1	1
2–4 h/day	276 (30.1)	447 (40.8)		1.57 (1.30, 1.90)	1.45 (1.19, 1.77)
>4 h/day	83 (9.1)	74 (6.8)		0.87 (0.62, 1.21)	0.77 (0.54, 1.10)
**Sleep duration, (** ***n*** **, %)**			<0.001		
<8 h	205 (22.4)	166 (15.2)		1	1
8–10 h	665 (72.6)	877 (80.1)		1.63 (1.30, 2.05)	1.62 (1.28, 2.06)
>10 h	46 (5.0)	52 (4.7)		1.40 (0.89, 2.18)	1.40 (0.88, 2.24)

^*^Analyses were adjusted for all variables presented in the table.

The dietary intake of children and adolescents based on physical activity adequacy is detailed in Table 3. A positive relation with adequate physical activity was observed for most variables related to the consumption of multiple servings of fruits, cereals, dairy products, meat, fish, legumes, nuts, and whole grains. However, for some food items, significant associations emerged only when moderate consumption (1–2 portions per day) was considered.

**Table 3:** Food group consumption of children and adolescents according to adequate level of physical activity (*n* = 2,011).

	**Physical activity**			**Physical activity**	
	**Inadequate**	**Adequate**	***p*** **-Value**	**OR (95% CI)**	**OR (95% CI)** ^*^
**Vegetables**, ***n*** **(%)**			<0.001		
Never	74 (8.1)	51 (4.7)		1	1
1–2 portion/d	770 (84.1)	882 (80.5)		1.66 (1.15, 2.41)	0.99 (0.65, 1.50)
≥3 portion/d	72 (7.9)	162 (14.8)		3.26 (2.08, 5.13)	1.33 (0.79, 2.23)
**Fruit**, ***n*** **(%)**			<0.001		
Never	74 (8.1)	19 (1.7)		1	1
1–2 portion/d	721 (78.7)	827 (75.5)		4.47 (2.67, 7.47)	3.07 (1.78, 5.30)
≥3 portion/d	121 (13.2)	249 (22.7)		8.01 (4.63, 13.88)	3.90 (2.15, 7.08)
**Cereals**, ***n*** **(%)**			<0.001		
Never	61 (6.7)	45 (4.1)		1	1
1–2 portion/d	794 (86.7)	907 (82.8)		1.55 (1.04, 2.30)	1.35 (0.89, 2.06)
≥3 portion/d	61 (6.7)	143 (13.1)		3.18 (1.95, 5.18)	1.96 (1.16, 3.32)
**Dairy**, ***n*** **(%)**			<0.001		
Never	230 (25.1)	174 (15.9)		1	1
1–2 portion/d	527 (57.5)	634 (57.9)		1.59 (1.27, 2.00)	1.42 (1.11, 1.81)
≥3 portion/d	159 (17.4)	287 (26.2)		2.39 (1.81, 3.15)	1.60 (1.19, 2.16)
**Meat**, ***n*** **(%)**			0.001		
Never	76 (8.3)	67 (6.1)		1	1
1–2 portion/week	503 (54.9)	538 (49.1)		1.21 (0.85, 1.72)	0.72 (0.48, 1.07)
≥3 portion/week	337 (36.8)	490 (44.7)		1.65 (1.15, 2.36)	0.92 (0.60, 1.40)
**Legumes**, ***n*** **(%)**			0.014		
Never	60 (6.6)	41 (3.7)		1	1
1–2 portion/week	631 (68.9)	764 (69.8)		1.77 (1.17, 2.67)	1.12 (0.71, 1.77)
≥3 portion/week	225 (24.6)	290 (26.5)		1.89 (1.22, 2.91)	0.96 (0.59, 1.56)
**Fish**, ***n*** **(%)**			<0.001		
Never	175 (19.1)	110 (10.0)		1	1
1–2 portion/week	611 (66.7)	759 (69.3)		1.98 (1.52, 2.57)	1.40 (1.04, 1.89)
≥3 portion/week	130 (14.2)	226 (20.6)		2.77 (2.01, 3.81)	1.25 (0.85, 1.83)
**Nuts**, ***n*** **(%)**			<0.001		
Never	449 (49.0)	304 (27.8)		1	1
1–2 portion/week	419 (45.7)	633 (57.8)		2.23 (1.84, 2.70)	1.87 (1.52, 2.30)
≥3 portion/week	48 (5.2)	158 (14.4)		4.86 (3.41, 6.93)	3.12 (2.12, 4.59)
**Whole grains**, ***n*** **(%)**			<0.001		
Never	290 (31.7)	277 (25.3)		1	1
1–2 portion/week	403 (44.0)	406 (37.1)		1.05 (0.85, 1.31)	0.87 (0.69, 1.10)
≥3 portion/week	223 (24.3)	412 (37.6)		1.93 (1.53, 2.44)	1.47 (1.14, 1.88)
**Sweets**, ***n*** **(%)**			0.043		
Never	88 (9.6)	72 (6.6)		1	1
1–2 portion/week	411 (44.9)	504 (46)		1.50 (1.07, 2.10)	1.30 (0.90, 1.87)
≥3 portion/week	417 (45.5)	519 (47.4)		1.52 (1.09, 2.13)	1.26 (0.88, 1.82)

^*^Analyses were adjusted for all variables presented in the table.

The average Y-HEI scores for children and adolescents with adequate physical activity compared to those with inadequate activity were 53.0 ± 12.0 and 50.9 ± 11.2, respectively (*p* < 0.001). After controlling for all previously identified significant background, dietary, and lifestyle variables related to the outcome, children and adolescents in the highest tertile of YEHI scores showed a 60% greater likelihood of engaging in adequate physical activity (OR = 1.60, 95% CI: 1.27, 2.01; Table 4). Notably, the diet quality score exhibited a linear relationship with the likelihood of achieving adequate sleep duration, with a 1-SD increase corresponding to an OR of 1.24 (95% CI: 1.13, 1.37).

**Table 4:** Association between diet quality of children and adolescents and adequate level of physical activity (*n* = 2,011).

	**Y-HEI, OR (95% CI)**			
	**T1**	**T2**	**T3**	**1-SD increase**
**Adequate physical activity**				
Model 1	1	1.26 (1.02, 1.55)	1.50 (1.21, 1.86)	1.20 (1.10, 1.31)
Model 2	1	1.26 (1.02, 1.56)	1.54 (1.24, 1.91)	1.22 (1.11, 1.33)
Model 3	1	1.25 (1.01, 1.56)	1.60 (1.27, 2.01)	1.24 (1.13, 1.37)

Model 1, unadjusted.

Model 2, adjusted for: age groups and parent's age.

Model 3, adjusted for: breakfast habit, eating out of home and eating at school.

## 4 Discussion

The objective of this study was to investigate the relationship between lifestyle choices and eating patterns linked to sufficient levels of physical activity in younger age groups within the Mediterranean area. Focusing on children and adolescents from five distinct nations, the research analyzed eating behaviors across these varied regions. This approach aimed to deliver an in-depth analysis of the elements affecting physical activity in the unique cultural and geographical settings of the Mediterranean. Findings highlighted key demographic traits of both parents and the young participants involved in the research, children with parents younger than 44 years old tend to exhibit a more adequate level of physical activity compared to those aged 45 and older. This association could be explained by several factors. Younger parents are often more physically active themselves, as they may have greater energy levels and fewer age-related health issues that could hinder participation in physical activities: as a result, they may serve as role models for their children, encouraging more active lifestyles (26). Moreover, younger parents might also be more engaged in modern trends and practices, such as enrolling their children in sports or outdoor activities. In contrast, older parents may have more established routines or face physical limitations that could affect their ability to model or encourage physical activity (27). Moreover, younger parents might prioritize fitness and active lifestyles, driven by cultural and societal expectations or a desire to model healthy behaviors for their children (28).

The relationship between employment status and physical activity levels revealed that unemployed parents exhibited significantly higher levels of physical activity compared to employed parents. This finding may be explained by the differences in time availability and daily routines between the two groups. Unemployed parents often have more discretionary time, which could allow them to engage in physical activities such as walking, exercising, or playing with their children (29). Conversely, employed parents may face greater time constraints due to work commitments and the demands of balancing professional and familial responsibilities, leaving them with fewer opportunities for regular physical activity. A similar result was found in a study where it was observed that parental employment is inversely associated with physical activity in girls, particularly when mothers are employed, as girls are less likely to engage in physical activity (30). In contrast, educational attainment appeared to have a positive association with physical activity adequacy, with parents holding higher levels of education demonstrating better adherence to recommended activity levels. This trend might be attributed to increased health literacy among more educated individuals, leading to a greater awareness of the benefits of physical activity and a stronger motivation to incorporate it into their lifestyle (31). Additionally, higher education levels often correlate with access to resources and environments conducive to physical activity, such as gyms, recreational facilities, or safe outdoor spaces (32). These findings highlight the complex interplay between socioeconomic factors and physical activity, suggesting that tailored interventions should address the unique barriers and facilitators associated with both employment status and educational attainment to promote active lifestyles across diverse populations.

Concerning lifestyle and eating behaviors of children and adolescents according to the adequate level of physical activity, were identified numerous significant findings, demonstrating how various dietary habits are closely associated with adequate levels of physical activity and, more broadly, a healthy lifestyle. In particular, a positive association was found between having breakfast and an adequate level of physical activity. This association could be explained by the fact that breakfast is the first meal of the day and provides the energy needed to face daily activities, including physical exercise (33). Eating a healthy breakfast can improve energy levels, concentration, and motivation, thus making it easier to engage in physical activity early in the day (34). Moreover, those who have breakfast tend to have a more regular routine and may be more aware of the importance of a healthy lifestyle, including physical exercise (35). An association was also observed between eating outside the home and an adequate level of physical activity. Specifically, individuals who reported eating meals outside the home, including those consumed at restaurants or other external settings, demonstrated a significant positive association with higher levels of physical activity. Eating outside the home may be a factor influencing the likelihood of engaging in sufficient physical activity. This could be explained by several factors. For instance, eating out may be linked to more structured meal times, which in turn may encourage individuals to maintain a routine that includes physical activity (36). Furthermore, certain external dining environments may promote healthier food choices, which may foster a more active lifestyle overall (37, 38). Similarly, a positive association was found for those who ate at school, reinforcing the connection between regular eating patterns and higher activity levels. School meals are often structured and provide balanced nutritional options, which could contribute to improved overall health and increased energy, facilitating physical activity (39). Additionally, the school environment itself may encourage physical activity through organized sports, physical education programs, and opportunities for active play during breaks (40). Both findings highlight the role of eating habits, particularly in structured settings such as eating out or at school, as significant factors in supporting an active lifestyle. These patterns suggest that external dining environments, whether at school or outside the home, may influence not only dietary choices but also broader lifestyle behaviors, such as engaging in regular physical activity. Understanding these connections can be important for developing interventions aimed at promoting physical activity and healthy eating in various social contexts (41).

Children and adolescents who reported a sleep duration (8–10 h) closest to the recommended one were significantly more likely to achieve adequate levels of physical activity, while those with excessive daily screen time (>4 h) were less likely to engage in adequate physical activity. These findings suggest a possible relationship between sleep patterns, screen time, and physical activity, where certain behaviors, such as adequate sleep and limited screen exposure, might encourage greater engagement in physical activities (42). One explanation could be that teenagers who sleep less may have more time during the day to engage in physical activities, as they are awake for a longer period (43). However, it's important to note that insufficient physical activity is generally associated with negative health outcomes, so this relationship may not be entirely beneficial in the long term (44). On the other hand, limiting screen time, especially in the context of sedentary activities like watching television or using digital devices, could promote a more active lifestyle (45). These findings are consistent with other studies: one in particular conducted in America, which investigated the relationship between physical activity, screen time, and the quantity and quality of sleep among U.S. adolescents aged 16–19. This study revealed that adolescents who meet the recommendations for physical activity might be more likely to experience insufficient sleep, as well as those who spend more time in front of screens (46). With fewer opportunities for passive activities, such as sitting for extended periods, teenagers may be more likely to participate in physical activities instead.

Regarding the association between an adequate level of physical activity of children and adolescents and the consumption of certain food groups, the study found that individuals who consumed fruit and vegetables, compared to those who did not, had an adequate level of physical activity. The association between an adequate level of physical activity and the consumption of fruits and vegetables can be explained through several biological and behavioral mechanisms linking nutrition to physical performance (47). Fruits and vegetables are foods rich in essential nutrients such as vitamins, minerals, fiber, and antioxidants, which support metabolism and muscle health, thus enhancing the ability to engage in regular physical activity (48). These foods also contribute to inflammation management and improved muscle recovery, both of which are crucial for physical exercise (49). Additionally, the presence of nutrients such as potassium and magnesium promotes proper muscle function and increased endurance during physical activity (50). Another important aspect is that a balanced diet, which includes fruits and vegetables, can have a positive impact on psychological well being and energy, stimulating motivation and the desire to engage in physical activity (51). This is particularly significant for children and adolescents, as it can help establish long-term healthy habits and foster a positive relationship with physical activity (52). By supporting both physical and mental health, a diet rich in fruits and vegetables can play a key role in promoting healthy development and reducing the risk of obesity, metabolic disorders, and other chronic health issues later in life (53). Therefore, the results suggest that the inclusion of these foods in the diet may not only improve overall health but also encourage physically active behavior, helping children and adolescents lead healthier and more active lives (54).

Similar results have been found for the consumption of cereals and whole grains because these foods, like fruits and vegetables, are rich in essential nutrients that support overall well being and promote physical activity. Whole grains, in fact, contain higher amounts of fiber, B vitamins, minerals (such as magnesium), and antioxidants compared to refined cereals (55). These nutrients are crucial for energy metabolism, muscle health, and inflammation regulation, all factors that positively influence the ability to engage in regular physical activity (56). The fiber present in whole grains also improves digestion and helps maintain stable blood sugar levels, preventing insulin spikes that could impair energy during physical activity (57). Furthermore, the consumption of whole grains has been shown to improve physical endurance and muscle recovery, as they provide slow-release energy, helping to sustain prolonged physical activity (58). Furthermore, it is not surprising to find that children and adolescents who engage in regular physical activity consume at least two servings of cereals per day, as they provide slow-release energy, which is essential for maintaining stable blood glucose levels during exercise (59). This type of sustained energy is particularly useful for prolonged or high-intensity physical activities, as it helps prevent sudden energy drops and improves overall endurance (60).

Regarding protein sources, significant results have been observed, particularly in relation to intermediate consumption, equivalent to 1–2 servings of meat, fish, and legumes. In general, children and adolescents who consume these foods more frequently appear to have a more adequate level of physical activity compared to those who consume them in limited or negligible amounts. The association between intermediate consumption of meat, fish, and legumes and an adequate level of physical activity can be explained by considering the crucial role that proteins play in supporting muscle mass and energy metabolism (61). The proteins provided by meat, fish, and legumes are essential for muscle building and recovery, two key aspects for individuals engaged in regular physical activity (62). Adequate protein intake also promotes muscle protein synthesis, which helps improve strength and endurance, thus optimizing physical performance (63). Furthermore, a balanced diet that includes a variety of protein sources can contribute to optimal energy management, improving the ability to perform physical activity regularly without early fatigue (64). This is particularly relevant for children and adolescents, as they are often involved in sports and other activities that require sustained energy and physical endurance. Therefore, intermediate consumption of these foods provides a sufficient amount of essential nutrients without overloading the body, optimally supporting physical activity and promoting an adequate level of activity (65). By focusing on balanced protein intake from meat, fish, and legumes, children and adolescents are better able to fuel their growing bodies and maintain the physical stamina needed for daily activities (52). Research suggests that individuals who follow a diet with balanced protein consumption from meat, fish, and legumes have more energy and endurance, facilitating engagement in physical activity (66).

The same result has also been found for dairy consumption, which, in addition to providing proteins, is also an excellent source of fats. Dairy products, particularly whole milk, cheese, and yogurt, contribute to energy balance, muscle repair, and overall metabolic health (25). Proteins in dairy products are of high biological value, meaning they contain all the essential amino acids needed for muscle growth, repair, and maintenance, which is critical for individuals engaging in physical activity (67). Additionally, the fats in dairy, especially saturated fats, provide a concentrated source of energy, which can support prolonged physical exertion and aid in recovery post-exercise (68). Moreover, the fats in dairy products play a role in the absorption of fat-soluble vitamins (such as vitamins A, D, E, and K), which are important for maintaining overall health and supporting various physiological functions, including those that regulate physical performance (69). These nutrients, combined with the proteins, help enhance endurance, strength, and recovery, which is why dairy consumption has been linked to higher levels of physical activity and improving motivation to be physically active (70).

Another interesting result, in line with what was described earlier, pertains to the consumption of nuts, as they are a rich source of healthy fats, protein, and fiber, which contribute to overall health and support physical activity. Nuts, such as almonds, walnuts, and pistachios, contain a high concentration of monounsaturated and polyunsaturated fats, which are beneficial for cardiovascular health and provide a sustained source of energy during physical exertion (71). In addition to their healthy fat content, nuts also provide essential nutrients like magnesium, potassium, and vitamin E, which support muscle function, electrolyte balance, and reduce oxidative stress, respectively (72). These factors play a role in improving endurance, muscle recovery, and overall exercise performance. Furthermore, the protein and fiber in nuts help with satiety and energy regulation, preventing fatigue and supporting sustained physical activity over longer periods (73).

All these results, when considered together, provide a clear picture of how a healthy lifestyle, which includes not only a balanced diet with the right intake of macro and micronutrients, is closely related to an adequate level of physical activity concerning the diet quality of children and adolescents and the association with an adequate level of physical activity. This emphasizes the importance of promoting healthy eating habits from a young age, as a balanced diet not only supports healthy growth but also encourages physically active behaviors (53). The integration of nutrition and physical activity can thus, play a crucial role in the development and maintenance of long-term health (74). Moreover, the fact that such associations emerge even in a younger population suggests the importance of early intervention, instilling habits that can reduce the risk of chronic diseases and improve quality of life (75).

To our knowledge, this research stands out for its ability to assess lifestyle factors related to physical activity among children and adolescents across five Mediterranean countries using a standardized methodology. Nonetheless, the findings must be interpreted while acknowledging certain limitations. The study's cross-sectional nature prevents the establishment of causal relationships. In fact, there is no actual causal relationship, while we hypothesize that the variables examined in the context of adequate physical activity are part of an overall cluster of healthy behaviors characterized by a more active lifestyle. Additionally, potential reporting bias may arise from the reliance on parental questionnaires to gather information about dietary patterns and eating behaviors. Hence, the reporting findings may suffer from involuntary recall bias or voluntary social desirability bias (to align with societal expectations of being health-conscious), although it should be systematically applied to all variables to truly affect the results (which is unlikely).

This study highlights the strong associations between healthy lifestyle behaviors and adequate physical activity levels in children and adolescents across the Mediterranean region. Regular breakfast consumption, frequent family meals, sufficient sleep (8–10 h), and a higher diet quality (rich on fruits, whole grains, fish, and legumes) were positively linked to greater physical activity engagement. Conversely, excessive screen time (>4 h/day) was associated with lower physical activity levels. These findings underscore the importance of balanced lifestyles and healthy dietary patterns in promoting well being. The study highlights the need for an integrated approach that combines dietary education and lifestyle interventions to encourage healthier habits in children and adolescents. Collaboration among parents, educators, and healthcare professionals is essential to create a supportive environment that fosters nutritious food choices and an active lifestyle. Such an environment, equipped with resources, educational programs, and practical strategies, can help families make informed decisions, ultimately improving dietary habits, physical activity levels, and long-term health outcomes in children.

## Data Availability

The raw data supporting the conclusions of this article will be made available by the authors, upon reasonable request.
